# Stable isotope data of Neolithic and Eneolithic populations in the Balkans, 6600 to 4000 BC

**DOI:** 10.1016/j.dib.2022.108114

**Published:** 2022-04-02

**Authors:** Aurélien Tafani, Cătălin Lazăr, Robert H. Tykot

**Affiliations:** a Laboratory for Archaeological Science and Technology (L.A.S.T.), Department of Anthropology, University of South Florida, 4202 E. Fowler Ave., SOC107, Tampa, FL 33620, USA; b ArchaeoSciences Division at Research Institute of the University of Bucharest (ICUB), University of Bucharest, 90 Sos. Panduri, 5th District, Bucharest, Romania

**Keywords:** Collagen (bone and dentine), Tooth enamel, Carbon, Nitrogen, Neolithic, Eneolithic, Palaeodiet

## Abstract

Stable isotopic ratios of carbon and nitrogen performed on collagen and tooth enamel offer invaluable insight into the diet of ancient populations. In the northern Balkans, most of these isotopic data have been collected as auxiliary information of radiocarbon dates, to correct a potential marine reservoir effect. In order to facilitate the access of the academic community to these data, we present a set of isotopic carbon and nitrogen ratios of human collagen samples for 188 individuals from 12 previously published sites together with hitherto unreleased data for 24 individuals from 4 sites from the Neolithic and Eneolithic period in Bulgaria and Romania. This collection also includes previously published carbon isotopic ratio measurements on tooth enamel of 34 individuals.

## Specifications Table

**Table:** 

Subject	Social Sciences – Archaeology
Specific subject area	Stable isotope analysis Bone and dentine collagen Tooth enamel Carbon Nitrogen Palaeodiet Subsistence economy
Type of data	Table Figure
How the data were acquired	A systematic literature review was conducted using Google Scholar, focusing on academic journals dedicated to archaeological science and archaeological research in Eastern Europe.
Data format	Raw
Description of data collection	An extensive literature review was conducted on material published before January 2021.
Data source location	Carbon and Nitrogen isotopic data from human samples dated of the Neolithic and Eneolithic period, between 6600 and 4000 BC. The remains were found in Bulgaria and Romania. Geographic coordinates are provided for each site, as indicated by the authors of the original scientific articles; if the coordinates were not mentioned in the scientific articles, the approximate location of the sites were extracted from Google Earth.
Data accessibility	Repository: IsoArcH (https://isoarch.eu/) [1] DOI of the dataset: 10.48530/isoarch.2021.015 Direct URL of the dataset: https://doi.org/10.48530/isoarch.2021.015 Data is available under the Creative Commons BY-NC-SA 4.0 license.

## Value of Data

• Stable isotope analysis has made a substantial contribution to our understanding of the passage from a way of subsistence based on hunting and gathering to the Neolithic mode of food production in other regions of Europe [2]; however, in the northern Balkans, this kind of analysis has not received much attention from the scientific community. This collection should demonstrate the usefulness of such data to explore this topic.
• Except for two studies [3,4], stable isotope analysis has been performed to correct a potential marine reservoir effect in radiocarbon dating. This collection includes the resulting data and should make them more accessible to researchers focusing primarily on the issue of ancient diet.
• This collection consists of data from sites located in an area that is now divided into two different countries, Bulgaria and Romania, but that belonged to the same material culture during the period of interest. Combining data from sites located in both states should offer a new perspective on the issue.

## 1 The Data

**Fig. 1:**
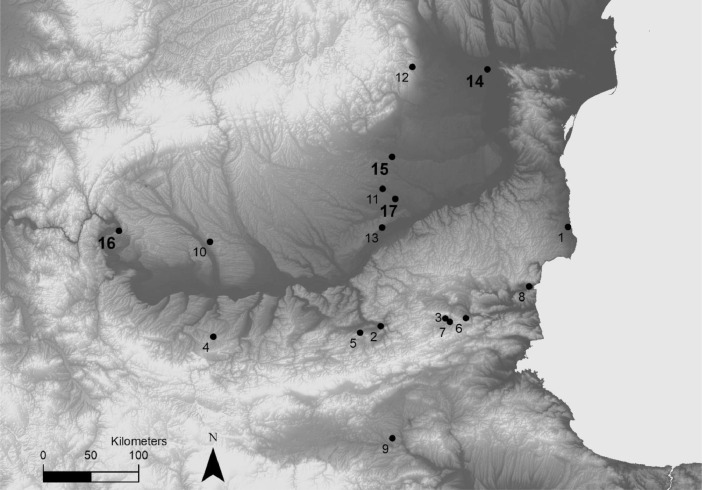
Map showing all the sites included in this work. The site IDs are provided in Table 1. The sites with hitherto unpublished data are indicated in bold with a larger font size.

This work collects carbon and nitrogen isotope data of Neolithic and Eneolithic populations (6600 to 4000 BC) from various scientific articles focused on chronological issues, where such isotopic data were mainly used to identify and correct a potential marine reservoir effect. This collection presents isotopic data on 246 individuals from 17 different sites, for a total of 288 entries. These entries consist of 257 pairs of carbon and nitrogen isotopic ratios measured on collagen from 212 individuals from 16 sites, including unreleased data on 24 individuals. These unpublished data were also used to correct potential marine reservoir effect on the radiocarbon measurements. Carbon isotopic ratio measurements on tooth enamel of 34 individuals from one site constitute the rest of the dataset. Auxiliary information regarding the location, time period and cultural affiliation of the sites is summarized in Table 1. The average δ^13^C (‰VPDB) and δ^15^N (‰AIR) values per period are given in Table 2. A map of the different sites is also included (Fig. 1).

**Table 1:** Site ID, site name, coordinates in WGS 84, time period, type of sample analyzed, number of individuals analyzed, and references for each site. The coordinates for the sites 1, 8, 11, and 13 were extracted from Google Earth.

Site ID	Site Name	Latitude	Longitude	Time Period	Type of Sample	Number of Individuals	References
1	Durankulak, Bulgaria	43.669	28.532	Early/Middle Eneolithic	Bone Collagen	77	[3,4]
2	Dzhulyunitsa, Bulgaria	43.161	25.883	Early Neolithic and Middle Eneolithic	Bone Collagen	3	[5]
3	Ivanovo, Bulgaria	43.099	26.719	Early Eneolithic	Dentin Collagen	1	[5]
4	Ohoden, Bulgaria	43.374	23.729	Early Neolithic	Bone Collagen	1	[5]
5	Samovodene, Bulgaria	43.140	25.604	Early Neolithic	Bone Collagen	1	[5]
6	Smyadovo, Bulgaria	43.060	26.980	Middle Eneolithic	Bone and Dentin Collagen	8	[6,5]
7	Sushina, Bulgaria	43.059	26.767	Middle Eneolithic	Dentin Collagen	3	[5]
8	Varna, Bulgaria	43.213	27.864	Middle Eneolithic	Bone Collagen	71	[7,3,4]
9	Yabalkovo, Bulgaria	42.100	25.750	Early Neolithic	Bone Collagen	1	[5]
10	Cârcea, Romania	44.263	23.900	Early Neolithic	Bone Collagen	1	[5]
11	Cernica, Romania	44.424	26.270	Middle Neolithic	Bone Collagen	20	[8]
12	Coțatcu, Romania	45.482	26.996	Middle Neolithic	Bone Collagen	1	[5]
13	Pietrele, Romania	44.068	26.156	Middle Eneolithic	Tooth Enamel	34	[9]
14	Brăilița, Romania	45.296	27.971	Early and Middle Neolithic	Bone Collagen	8	This work
15	Dridu, Romania	44.698	26.477	Middle Eneolithic	Bone Collagen	3	This work
16	Ostrovul Corbului, Romania	44.515	22.742	Middle Eneolithic	Bone Collagen	10	This work
17	Popeşti, Romania	44.305	26.402	Early Eneolithic	Bone Collagen	3	This work

**Table 2:** Average δ^13^C (‰VPDB) and δ^15^N (‰AIR) values per period, with number of individuals analyzed for each period. The values for one individual from Brăilița and for one individual from Ostrovul Corbului, which did not pass the quality control criteria established for this study, are not included in the calculation.

Period	n	Average δ^13^C (‰VPDB)	Range δ^13^C (‰VPDB)	Average δ^15^N (‰AIR)	Average δ^15^N (‰AIR)
Early Neolithic	12	–18.9 ± 1.2	–20.6 to –17.2	14.5 ± 4.8	9.2 to 20.8
Middle Neolithic	24	–20.1 ± 0.6	–20.9 to –17.4	10.4 ± 1.7	9.2 to 18.7
Early Eneolithic	78	–19.1 ± 0.8	–20.3 to –12.3	9.4 ± 1.1	7.6 to 13.7
Middle Eneolithic	98	–19.3 ± 0.5	–21 to –17.8	10.3 ± 1.1	8 to 14.2

## 2 Research Design

Data were collected from sites located on the territories of the present-day countries of Bulgaria and Romania. A systematic literature review was conducted on Google Chrome using Google Scholar, focusing on academic journals dedicated to archaeological science and archaeological research in Eastern Europe, using keywords such as Romania, Bulgaria, Neolithic, Eneolithic, Copper Age, Stable Isotopes, and Radiocarbon Dating. The current border between these two countries largely corresponds with the Danube River; however, it was not the case during Prehistory, as most archaeological cultures existed on both banks of the river. We chose to only present data that could be ascribed to the Neolithic and Eneolithic periods, as most authors consider that the period between 6600 and 4000 BC constitutes a consistent cultural block [10]. Although they refer to similar cultural phenomena, Bulgarian and Romanian chronological divisions differ. In this work, for convenience, we chose to adopt the Romanian chronology that consists of two periods, the Neolithic and the Eneolithic, each of them being subsequently subdivided into an early and a middle phase (Table 3). In addition, radiocarbon dates are reported when they are available.

**Table 3:** Chronological framework used in this work.

Period	Phase	Duration
Neolithic	Early	6600 to 5500 BC
	Middle	5500 to 5000 BC
Eneolithic	Early	5000 to 4500 BC
	Middle	4500 to 4000 BC

Whenever they were mentioned, we indicate the geographic coordinates of the sites as they appeared in the related scientific articles. For cases where such information was missing, we provide approximate locations extracted from Google Earth. The altitude and the distance to the modern coastline were also extracted from Google Earth. Finally, the coordinates from the unpublished sites are those published in Lazăr et al. [10].

The majority of the source scientific articles are concerned with radiocarbon dating rather than with ancient diet, with information about the collagen extraction and sample analysis methods sparse. Only Honch et al. [3] and Higham et al. [7] provide details regarding the methodologies they followed, which were published in Bronk Ramsey et al. [11] and Brock et al. [12], respectively.

Regarding the quality control of the collagen samples, we followed Ambrose's [13] recommendations and rejected samples that had a C/N ratio below 3.2 or above 3.6. Based on this criterion, we did not include the data available for one sample from Brăilița and for one sample from Ostrovul Corbului. Whenever possible, other useful indicators of collagen integrity [14] are also reported.

## Ethics Statements

This study does not involve any modern human or animal subject.

## CRediT authorship contribution statement

**Aurélien Tafani:** Conceptualization, Methodology, Writing – original draft. **Cătălin Lazăr:** Writing – review & editing, Visualization, Funding acquisition. **Robert H. Tykot:** Writing – review & editing.

## Declaration of Competing Interest

The author declares that they have no known competing financial interests or personal relationships which have or could be perceived to have influenced the work reported in this article.
